# Heart rate variability as a predictor of intraoperative autonomic nervous system homeostasis

**DOI:** 10.1007/s10877-024-01190-x

**Published:** 2024-07-13

**Authors:** Ole C. Keim, Lennart Bolwin, Robert E. Feldmann, Jr., Manfred Thiel, Justus Benrath

**Affiliations:** 1 Department of Anesthesiology, Pain Center, Medical Faculty Mannheim, Heidelberg University, Theodor-Kutzer-Ufer 1-3, 68167 Mannheim, Germany; 2 German Economic Institute, Data Science Consultant, Konrad-Adenauer-Ufer 21, 50668 Köln, Germany

**Keywords:** Heart rate variability, Sevoflurane, Autonomic nervous system, Depth of anesthesia, Narcotrend index, Minimum alveolar concentration

## Abstract

The aim of the proof-of-concept study is to investigate the level of concordance between the heart rate variability (HRV), the EEG-based Narcotrend Index as a surrogate marker for the depth of hypnosis, and the minimal alveolar concentration (MAC) of the inhalation anesthetic sevoflurane across the entire course of a surgical procedure. This non-blinded cross-sectional study recorded intraoperative HRV, Narcotrend Index, and MAC in 31 male patients during radical prostatectomy using the Da-Vinci robotic-assisted surgical system at Mannheim University Medical Center. The degree of concordance was calculated using repeated measures correlation with the R package (rmcorr) and presented using the rmcorr coefficient (rrm). The Narcotrend Index correlates significantly across all measures with the time-dependent parameter of HRV, the standard deviation of the means of RR intervals (SDNN) (rrm = 0.2; *p* < 0.001), the frequency-dependent parameters low frequency (LF) (rrm = 0.09; *p* = 0.04) and the low frequency/high frequency ratio (LF/HF ratio) (rrm = 0.11; *p* = 0.002). MAC correlated significantly negatively with the time-dependent parameter of heart rate variability, SDNN (rrm = -0.28; *p* < 0.001), the frequency-dependent parameter LF (rrm = -0.06; *p* < 0.001) and the LF/HF ratio (rrm = -0.18; *p* < 0.001) and the Narcotrend Index (rrm = -0.49; *p* < 0.001) across all measures. HRV mirrors the trend of the Narcotrend Index used to monitor depth of hypnosis and the inhibitory influence of the anesthetic sevoflurane on the autonomic nervous system. Therefore, HRV can provide essential information about the homeostasis of the autonomic nervous system during general anesthesia. DRKS00024696, March 9th, 2021.

**Supplementary Information:**

The online version contains supplementary material available at 10.1007/s10877-024-01190-x.

## Introduction

To date, depth of general anesthesia is monitored using surrogate parameters such as blood pressure, heart rate and the minimal alveolar concentration (MAC) of the inhalational anesthetic. A clear distinction between the depth of hypnosis and analgesia is not possible using these parameters. First approaches to monitor depth of anesthesia in terms of recording the hypnosis-level and not the analgesic-level were conducted using noninvasive continuous electroencephalography (EEG). The Narcotrend Index allows now a coarse assessment of central sedation during surgery [1]. Knowledge of depth of hypnosis is essential in order to prevent patient awareness, which is still is a relevant problem with an incidence of 0.6% during general anesthesia [2]. Conversely, excessive depth of hypnosis has been shown to be independently associated with prolonged patient recovery and increased postoperative 1-year mortality [3, 4]. However, EEG analysis cannot be utilized under all circumstances [5]. Furthermore, monitoring surrogate markers for the effects of general anesthesia on the CNS by EEG remains incomplete, since the inhaled agents exert a strong inhibitory influence on the autonomic nervous system (ANS) as well [6]. Of particular note, perioperative mortality and morbidity are directly associated with the activity level of the patient’s ANS in terms of the exaggerated stress response of the postaggression metabolism [7–9]. Here, the most rapid responses are seen in proinflammatory stimuli of the sympathetic branch of the ANS [10, 11]. In contrast, afferent vagal nerve fibers facilitate important rapid anti-inflammatory responses. [12] The delicate balance between both effector systems in the ANS can be assessed by heart rate variability (HRV) measured via conventional ECG [13]. HRV is not only directly negatively related to the important inflammatory markers tumor necrosis factor α (TNF-α) and interleucin 6 (IL 6), but also reflects inhibition of the inflammatory response by the vagal nerve [12, 14]. It has been shown as early as 1993 that intraoperative stress by means of surgical stimulation provokes decrease in HRV [15]. Since HRV can serve as an indicator of brainstem activity and thus ANS function, a promising method has become available to complement EEG measurements with the goal of maintaining autonomic homeostasis during general anesthesia [16–18]. However, the pioneering studies of the level of concordance between EEG and HRV have mostly been performed on awake patients [19–21], under experimental conditions [22] or have been performed during induction of general anesthesia [17, 18]. To our knowledge, the level of concordance between EEG and HRV during general anesthesia has not been adequately studied yet.

The aim of the current study is to investigate the level of concordance between the HRV parameters (SDNN, RMSSD, HF, LF, LF/HF ratio), the EEG-based Narcotrend Index as a surrogate marker for the depth of hypnosis, and the MAC of the inhalation anesthetic sevoflurane across the entire course of a surgical procedure. The study does not aim to assess the analgesic-level. It is hypothesized that HRV parameters are concordant with the Narcotrend Index and consequently mirror the same trends. It is also hypothesized that HRV parameters are concordant with MAC and therefore provide information about the influence of sevoflurane on the homeostasis of the autonomic nervous system during surgery under general anesthesia.

## Materials and methods

This cross-sectional study collects data in the perioperative setting. After approval by the Ethics Committee II of the University of Heidelberg, Germany (2020–550 N, June 16th, 2020), 34 male patients underwent radical prostatectomy using the Da-Vinci robotic-assisted surgical system (Intuitive Surgical Inc., California, USA) after written and verbal informed consent regarding study-related measures. The study was entered into the German Clinical Trials Register (DRKS00024696, March 9th, 2021). Patients gave written consent to data collection one day before surgery. Exclusion criteria included age less than 18 and greater than 85 years, diabetes mellitus, ASA status > 2, use of medications affecting the autonomic nervous system, e.g., β-blockers, antiarrhythmics, and atropine, implantation of a pacemaker, and cardiac arrhythmia. A prior calculation of the sample size was not carried out due to the exploratory design of the study.

### Data collection

**Fig. 1:**
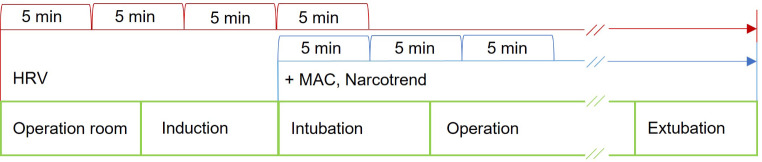
Study protocol. HRV, MAC, and Narcotrend Index were recorded continuously at 5-minute intervals. Abbreviations *HRV = Heart rate variability; MAC = Minimal alveolar concentration

The informational consent and examination for potential exclusion criteria took place in the anesthesiologic premedication clinic and included the recording of a 12-lead ECG. No medications were given during premedication. Immediately after patient’s appearance in the induction room, intravenous and intraarterial access and standard monitoring of vital parameters were initiated. This included measurement of blood pressure by Riva-Rocci, invasive arterial blood pressure measurement, oxygen saturation by digital infrared spectral oximetry, temperature by esophageal probe, and 3-lead ECG. Simultaneously, the study-related acquisition of HRV started. HRV was recorded at 5-minute intervals throughout the duration of general anesthesia. The subsequent induction of anesthesia into a balanced general anesthesia maintained with sevoflurane was performed according to the clinic’s own SOP with sufentanil (0.2 µg/kgBW), propofol (titrated 1.5-2-3 mg/kgBW), and rocuronium (0.6 mg/kgBW). After intubation, recording of the age adjusted MAC and Narcotrend Index began using the Narcotrend monitor and the Atlan A350 (Drägerwerk AG & Co. KGaA, Lübeck, Germany). The Narcotrend Index and MAC were recorded manually on a spreadsheet in five-minute intervals. Their acquisition was continued synchronously with HRV in five-minute intervals until extubation. The study protocol is visualized in Fig. 1.

#### About HRV and narcotrend

Heart rate variability was recorded and analyzed using the HRV scanner (BioSign GmbH, Ottenhofen, Germany) according to the recommendations of the 1997 Task Force [23]. The QRS complexes of cardiac Depolarization were recorded in a bipolar setup with a sampling rate of 500 Hz. The acquired ECG was then examined for artifacts. In a first step, artifact elimination is performed using a software-based high-pass and low-pass filter. The filters are configured individually for each subject based on their resting HR. In a second step, the automatically detected R-waves are manually checked for plausibility and falsely detected artifacts were deleted. On the contrary, unrecognized R-waves are manually integrated into the calculation of the HRV. These steps were performed separately for each five-minute interval. Subsequently, the variability of R-R intervals was evaluated using statistical methods, Fast Fourier Transform, autoregression, and regressive spectral analysis. Data analysis yielded the time-domain parameters “Standard deviation of NN intervals” (SDNN) and the “Root Mean Square of successive differences” (RMSSD). SDNN is interpreted to represent the global variability of the heart rate and thus reflects the regulatory capacity of the ANS [23]. The RMSSD mainly epitomizes the parasympathetic influence on the heart function [24]. The frequency-related parameters consist of the higher frequency (HF; 0.15–0.4 Hz) and the lower frequency (LF; 0.04–0.15 Hz) portions in absolute values with the unit milliseconds squared [23]. HF-related changes in heart rate are largely caused by the parasympathetic nervous system [25, 26]. LF is much less specific to a component of the ANS [26]. Consequently the LF component of HRV is determined by influences from both the sympathetic and parasympathetic systems [27]. Despite the low discriminatory power of LF, the synopsis of LF and HF allows for an assessment of sympathovagal balance. [28]

To determine depth of hypnosis to avoid awareness and excessive depth, the CNS activity was measured by electroencephalographic recording of cortical potential fluctuations using the Narcotrend monitor (MonitorTechnik, Bad Bramstedt, Germany). [29] The Narcotrend monitor records the patient’s EEG via three commercially available ECG electrodes attached to the forehead issues the numerical Narcotrend Index (between 0 (electrical silence) and 100 (awake)) reflecting the momentary depth of hypnosis. A detailed overview of the underlying calculations is given by Kreuer, et al. [30].

### Statistical analysis

Statistical analyses were performed using IBM SPSS Statistics 27 software (SPSS Inc., Chicago, IL) and the repeated measures correlation (rmcorr) package for the free software environment R. Descriptive statistics are expressed as median between the 25th and 75th percentile. The correlation between the Narcotrend Index and MAC was calculated using rmcorr [31]. In contrast to common regressions or correlations, rmcorr does not violate the assumption of normal distribution and independence of observation variables. Furthermore, the method does not require accumulation or averaging of data. This allows the calculation of linear correlations between two parameters with repeated measurements and controls at the same time for inter-subject confounding. Similar to the Pearson correlation coefficient (r), the rmcorr coefficient (rrm) ranges from − 1 to 1, with 1 indicating the strongest possible positive correlation. To enhance external validity and to control for intra-subject confounding, we used robust standard errors, p-values and confidence intervals. All statistics were two-tailed with a 95% confidence interval. *p* < 0.05 was assumed to represent significance for all tests.

## Results

**Fig. 2:**
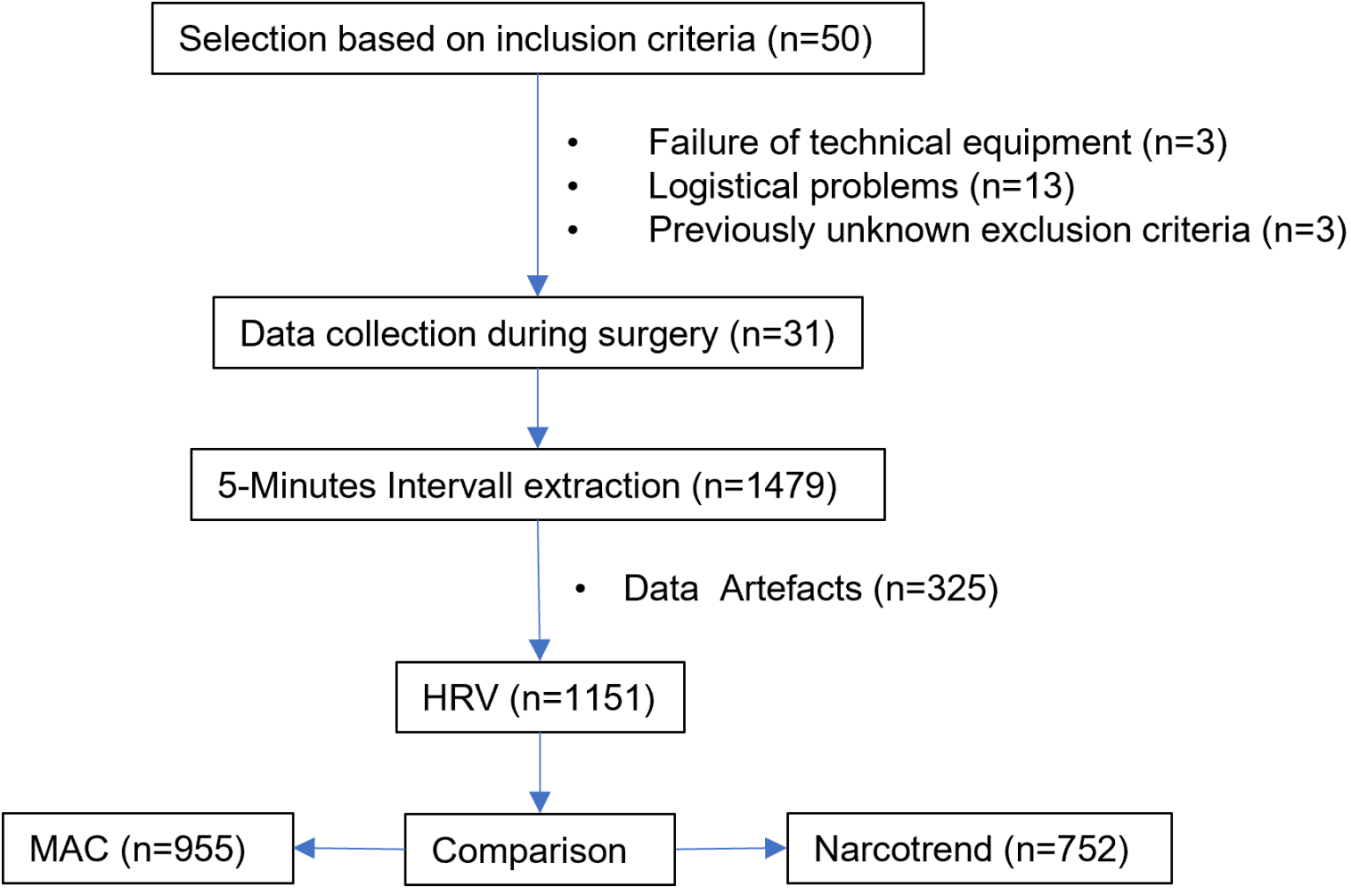
Development of the study population and data processing. *Abbreviations* *MAP = Mean arterial pressure; HRV = Heart rate variability; MAC = Minimal alveolar concentration

Based on 50 patients who met the inclusion criteria and gave written informed consent for study-related data collection during their scheduled radical prostatectomy using the Da-Vinci robotic-assisted surgical system between August 27, 2020, and March 13, 2021, 31 patients were followed during surgery. Nineteen patients could not be included in the study because of logistical difficulties, such as postponed (*n* = 6) or early (*n* = 4) surgery dates and failure of the technical equipment (*n* = 3), or due to subsequent fulfillment of an exclusion criterion, such as new-onset atrial fibrillation (*n* = 2), application of β-blockers (*n* = 4). For the 31 included patients, a total of 1479 5-minute intervals were collected during an average operative time of 227 min. Of those, 325 5-minute intervals, corresponding to 22% of the data volume, had to be excluded due to artifacts and technical problems yielding 1151 5-minute intervals for which the HRV parameters could be secured free of artifacts. Of those, 1151 5-minute intervals, 752 could be used for the computation of the degree of agreement with the Narcotrend Index. For the computation of the degree of agreement with the MAC, 955 5-minute intervals could be used. The difference in number of intervals recorded resulted primarily from the study protocol design. The acquisition of HRV, MAC and Narcotrend Index started one after the other, thus yielding different numbers of value pairs. A graphical representation of the composition of the measurement intervals can be found in Fig. 2. Demographic characteristics of the included patient population are depicted in Table 1.

**Table 1:** Demographic data *Abbreviations* * BMI = body Mass Index; ASA = classification according to American Association of Anesthesiology

Parameter	Mean	Range
Age (years)	63	(43–78)
BMI (kg/m^2^)	26	(20.6–33.6)
ASA	2	(2–3)
Duration of Surgery (min)	225	(145–310)

**Fig. 3:**
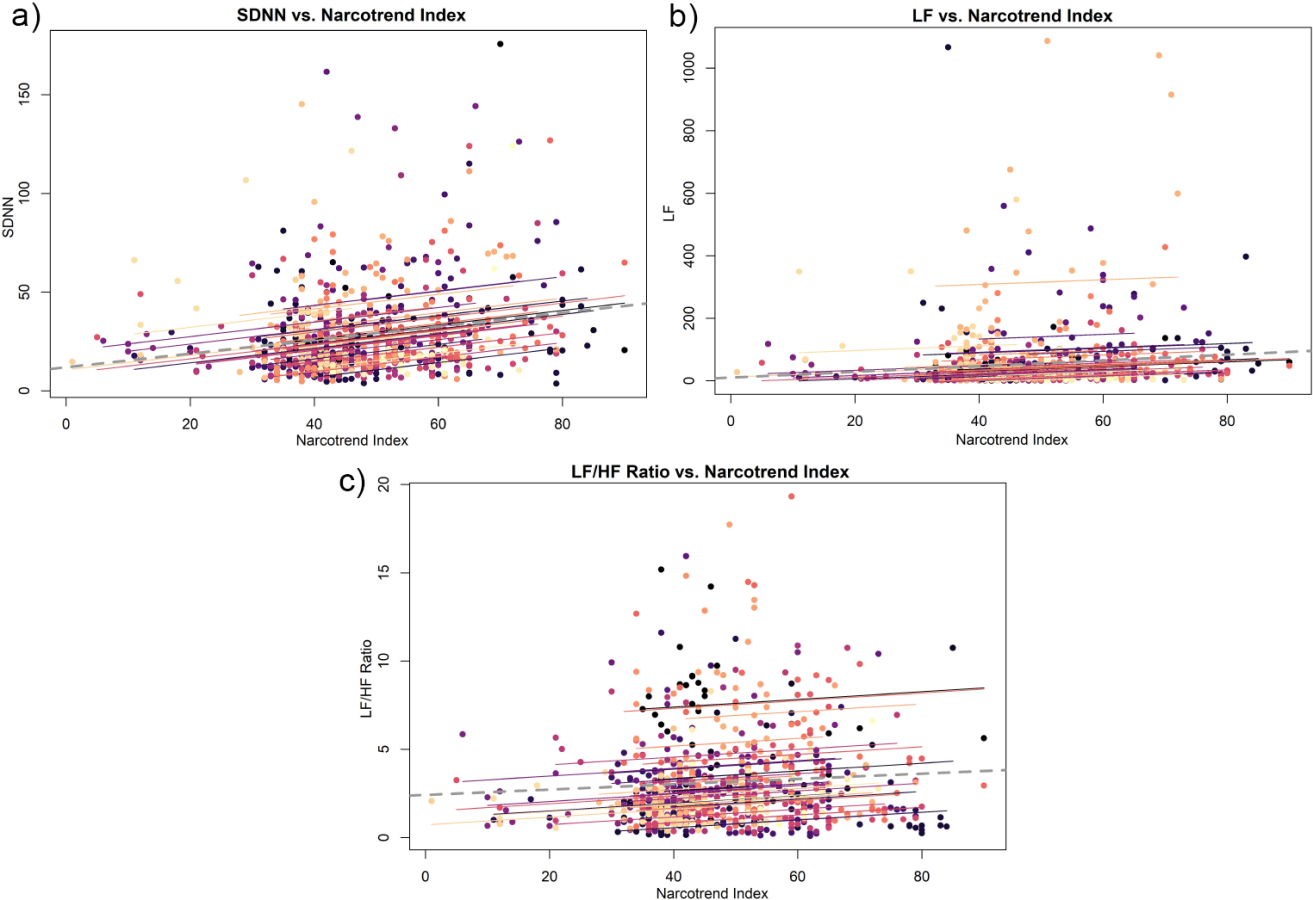
Correlation between Narcotrend Index and HRV parameters. *Abbreviations* * SDNN = Standard deviation of NN intervals; LF = Low frequency power; LF/HF Ratio = Ratio of low and high frequency power; Coloured dots = Patients

The analysis of the results shows that Narcotrend Index correlates significantly positively with the time- and frequency dependent parameters of HRV across all measured values. For the time-dependent parameters, this correlation could only be demonstrated for the SDNN (rrm = 0.2; 95% CI [0.10; 0.30], *p* ≤ 0.001). The RMSSD (rrm = -0.01; 95% CI [-0.08; 0.04], *p* = 0.64), on the other hand, does not correlate with the Narcotrend Index. The analysis of the frequency-dependent parameters of HRV revealed a significant correlation of LF (rrm = 0.09; 95% CI [0.004; 0.18], *p* = 0.04) with the Narcotrend Index. HF (rrm = -0.05; 95% CI [-0.14; 0.03], *p* = 0.27) on the other hand, does not correlate with the Narcotrend Index when assessed in isolation. However, if we consider the relationship of both parameters to each other, we can show a significant correlation of the LF/HF ratio (rrm = 0.11; 95% CI [0.04; 0.18], *p* = 0.002) with the Narcotrend Index. Narcotrend Index correlations are graphically shown in Fig. 3a-c.

**Fig. 4:**
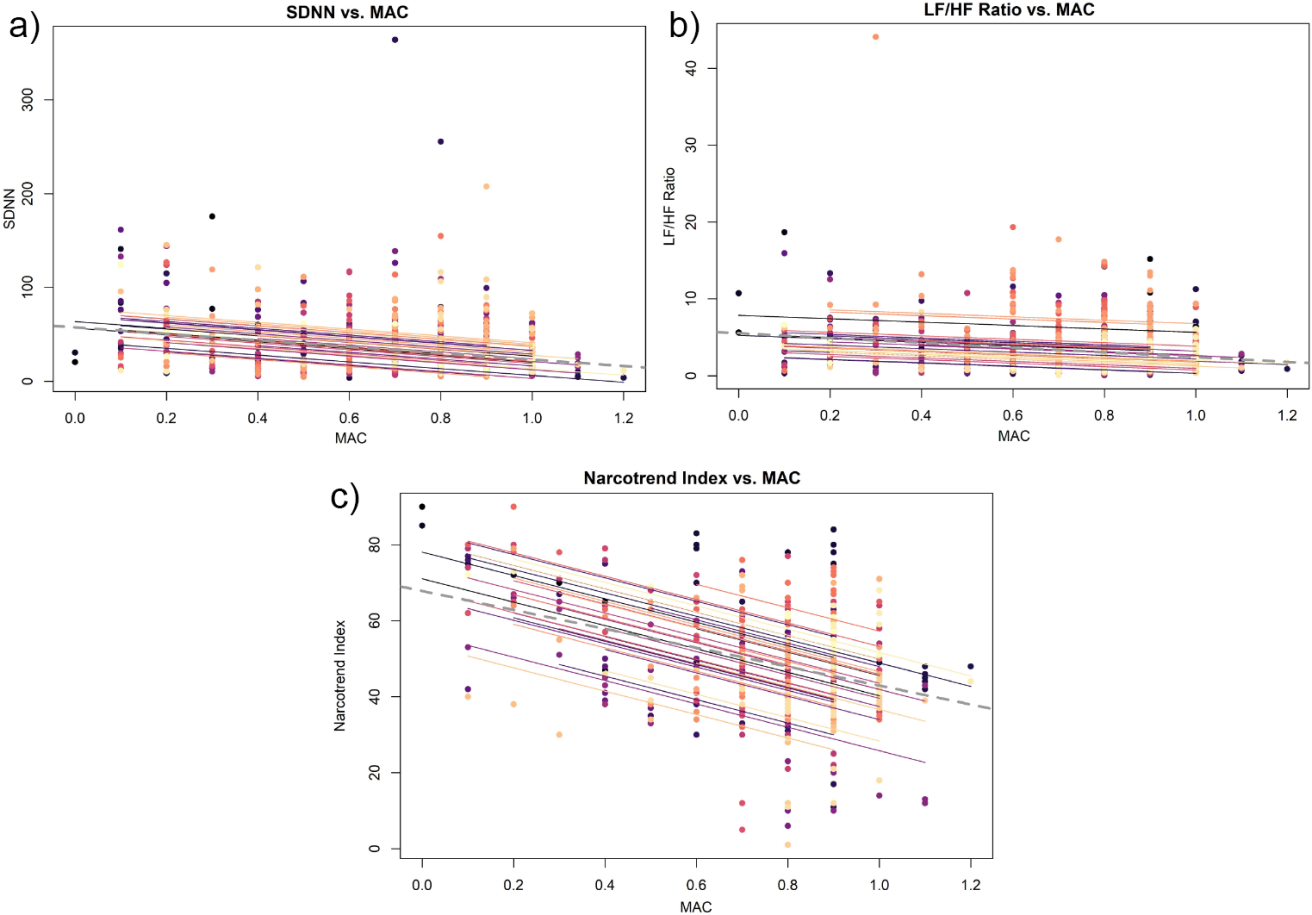
Correlation between MAC and HRV Parameters. *Abbreviations* * MAC = Mean alveolar concentration; SDNN = Standard deviation of NN intervals; LF/HF Ratio = Ratio of low and high frequency power; Coloured dots = Patients

The analysis of the results although shows a significantly negatively correlation between the MAC and the time- and frequency dependent parameters of HRV across all measures. For the time-dependent parameters, this correlation could again only be proven for the SDNN (rrm=-0.28; 95% CI [-0.36; -0.21], *p* ≤ 0.001), and not for the RMSSD (rrm=-0.00; 95% CI [-0.04; 0.03], *p* = 0.76). Also, MAC correlated significantly negatively across all measures with the relation of the frequency-dependent parameter of HRV to each other, the LF/HF ratio (rrm = -0.18; 95% CI [-0.25; -0.10], *p* ≤ 0.001) and with the LF (rrm = -0.06; 95% CI [-0.09; -0.02], *p* = < 0.001) when assessed in isolation. But not with HF (rrm =-0.01; 95% CI [-0.05; 0.01], *p* = 0.33) Graphic representations of the correlations with MAC are shown in Fig. 4a-c.

In addition, the Narcotrend Index correlated significantly negatively with MAC across all measures (rrm = -0.49; 95% CI [-0.53; -0.45], *p* < 0.001). A tabulation of the correlations between Narcotrend, MAC, and the HRV parameters can be found in Table 2.

**Table 2:** Correlation chart. *Abbreviations* *Green colour = positive correlation; red colour = negative correlation; MAC = Mean alveolar concentration; SDNN = standard deviation of NN intervals; RMSSD = Root Mean Square of successive differences; HF = high frequency power; LF = low frequency power; LF/HF ratio = ratio of low and high frequency power

	Narcotrend Index	MAC
Narcotrend Index		r_rm_ = -0.49 *p* ≤ 0,001
MAC	r_rm_ = -0.49 *p* ≤ 0,001	
SDNN	r_rm_ = 0.20 *p* ≤ 0,001	r_rm_ = -0.28 *p* ≤ 0,001
RMSSD		
HF		
LF	r_rm_ = 0.09 *p* = 0,04	r_rm_ = -0.06 *p* ≤ 0,001
LF/HF Ratio	r_rm_ = 0.11 *p* = 0,002	r_rm_ = -0.18 *p* ≤ 0,001

## Discussion

The central finding of the study is that HRV mirrors the trend of the Narcotrend Index used to monitor depth of hypnosis and the inhibitory influence of the anesthetic sevoflurane on the autonomic nervous system. To date, the assessment of the homeostasis of the ANS remains unattainable with the currently used cardiovascular parameters, the Narcotrend Index or MAC. The study also demonstrates that HRV not only provides information about the activity of the ANS but allows conclusions about central sedation during general anesthesia. The study thus provides new support that the technically more complex recordings required for monitoring the depth of hypnosis during general anesthesia using the Narcotrend Index might be usefully supplemented by the more simple recording of HRV.

Depth of hypnosis, in addition to monitoring cardiovascular parameters, is most commonly monitored using EEG-based techniques [29]. These surrogate parameters, however, reflect the influences of general anesthesia on the CNS and do not mirror effects on the ANS [17]. Recent studies on the impact of anesthesia on the ANS validated HRV as a suitable method for assessing the depth of hypnosis [18, 32]. In a pilot study, Zhan, et al. [5] showed that the time and frequency parameters of HRV facilitate the assessment of anesthesia across a wide ranges of levels. So far, the level of concordance of EEG and HRV has been tested on awake patients [19–21], under experimental conditions [22] or performed during the induction of general anesthesia [16–18]. But it has not been adequately studied throughout general anesthesia. EEG-Analysis has only been compared to the HRV-based Mdoloris Anaesthesia Nociception Index (ANI) monitor (Mdoloris Medical Systems, Lille, France) [33]. The ANI exclusively uses the frequency-dependent HRV parameter HF to monitor analgesia [34]. The level of concordance between the Narcotrend Index as a marker for depth of hypnosis and the other frequency- and time-dependent HRV parameters, however, has not yet been investigated. This is of particular importance as a selective recording of the two components of depth of anesthesia, hypnosis and analgesia, using specific HRV parameters would be desirable. The design of this study is intended to explicitly evaluate the depth of hypnosis using HRV.

The current study demonstrates a significant positive correlation of the HRV parameters SDNN, LF, and LF/HF ratio with the Narcotrend Index. Thus, in a clinical context, both Narcotrend Index and the HRV parameters decrease with increasing depth of narcosis. While correlations are not proof of causality, a directional association of SDNN, LF, and LF/HF ratio with the Narcotrend Index can be concluded. SDNN represents the global variability of the heart rate [23] and is associated with the general influence of the ANS on cardiac function [35]. Therefore, it can be concluded that increasing depth of anesthesia entails a decline in the overall regulatory capacity of the ANS. This is consistent with previous studies which have demonstrated a sharp decrease of HRV during induction of general anesthesia [17, 18]. Furthermore the clinical data acquisition in this study, supports the advantages in assessing HRV-based depth of hypnosis described by Zhan, et al. [5] and facilitates validation of the HRV parameters by measuring them perioperatively in the context of the established methods. Further studies will be necessary to compare the predictive accuracy of processed EEG with a predictive model using EEG variables and HRV parameters. Further research should capture inadvertent “light anesthetic” events in addition to the naturally occurring changes in anesthetic depth observed in this study.

Beside EEG-based monitoring depth of hypnosis, the MAC of the inhalational anesthetic sevoflurane, regardless of considering only one component of general anesthesia, is often the only marker used to estimate depth of anesthesia [36]. The complex central mechanisms of inhaled anesthetics as well as the agents action on the ANS are still poorly understood. So far, the MAC of the inhalation anesthetic has been compared to HRV parameters in canine research only [37]. Other studies have examined the effect of sevoflurane on HRV in humans [38–40]. Studies investigating the degree of concordance between MAC and frequency- and time-dependent HRV during general anesthesia in humans, however, are lacking. The current study yielded a negative correlation of the HRV parameters SDNN, LF and LF/HF ratio with the MAC of sevoflurane. As mentioned, SDNN represents the expression of the global variability of the heart rate [23]. The negative correlation between SDNN and MAC can be explained by a general attenuation of the ANS tone with increasing sevoflurane concentrations. Furthermore, the results of this study confirm the scientific consensus that sevoflurane leads to a decrease in LF and the LF/HF ratio [16, 38–40], but no correlation was found between MAC and RMSSD and HF. It is assumed, that LF allows important conclusions about the sympathovagal balance of the ANS only if embedded in the LF/HF ratio [28], whereas the HF and the RMSSD specifically reflect the activity of the parasympathetic nervous system [25, 26]. Hence, sevoflurane inhibits sympathetic activity without affecting parasympathetic activity, an observation supporting the assumption that sevoflurane may be associated with a cardioprotective effect [41]. In animal experiments, this effect was shown to be mediated per intracellular signaling cascades, vascular regulation, and post-transcriptional modification [42, 43]. Results allow for the important conclusion that HRV represents the inhibitory influences of sevoflurane on the ANS, in addition to mirroring depth of hypnosis. The standard monitoring parameters currently used do not provide this feature [44]. 

Furthermore, above reflections initiate discussions about whether HRV recordings may usefully supplement EEG recordings with respect to clinically valid and reliable markers for assessing depth of hypnosis by providing essential information about the homeostasis of the ANS. There is growing evidence that EEG-based monitoring of anesthetic depth leads to a reduction of the amount of anesthetic used and thus to a more rapid recovery after anesthesia [45–47]. Further studies will be necessary to show that HRV may meet this demand as well. Most importantly, the preoperative detection and reduction of postoperative mortality is not possible by EEG-based measurement of anesthesia depth [48]. In contrast, there is evidence that HRV reliably detects potential postoperative mortality [49–51]. Stein, et al. [49] validated the HRV as an independent risk factor for mortality after cardiovascular events in 740 patients. A low HRV may exhibit prognostic qualities with regard to long-term morbidity and mortality in surgical patients [50]. Filipovic, et al. [51] showed that a preoperative LF/HF ratio below two served as the best predictor for two-year all-cause mortality in 167 patients with coronary artery disease. Based on the results of this study, HRV not only provides clinically relevant orientation concerning the activity of the CNS but also enables the assessment of homeostasis of the ANS during general anesthesia. The informational value of HRV for the clinician thus exceeds the yield when recording EEG alone.

This study is subject to limitations. Firstly, discussions about the interpretation of the magnitude of the correlation between HRV, Narcotrend index, and MAC are necessary. Hemphill [52] extended the benchmarks of Cohen [53] for a more realistic approach in behavioral science. According to Hemphill [52] published empirical guidelines, correlation coefficients of < 0.20 are in the lower third, those of 0,2 - < 0,3 are in the middle third and those of > 0,3 are in the upper third. According to this approach, the correlation between HRV, Narcotrend Index, and MAC should be classified in the middle third. Data acquisition in this study could only be accomplished for 31 of the 50 patients originally providing their informed consent. This reduction in sample size downgrades the validity of the results and could have reduced the magnitude of correlation. However, compared with other studies with a similar question, the number of subjects in the sample examined in this study is not significantly lower [17, 54]. Also, only patients with an American Society of Anesthesiologists physical status class 1 and 2 were included. Consequently, the study cannot draw conclusions about the associations in patients with more severe preexisting conditions. Finally, due to a lack of interface between the different hardware components, the Narcotrend Index and MAC were manually transferred to an Excel spreadsheet - a method which is a priori more error-prone than direct digital recording. For future studies, it is desirable to improve cooperation between the Hardware manufacturer and the researchers to enable digital processing of the data. HRV data were recorded digitally.

In conclusion, HRV mirrors the trend of the Narcotrend Index used to monitor depth of hypnosis. Also, HRV reflects the inhibitory influence of the anesthetic sevoflurane on the autonomic nervous system and provides essential information about the homeostasis of the ANS during general anesthesia. Given the simplicity of HRV implementation in clinical practice, the validation of the results in larger clinical trials is encouraged. Future studies should examine intraoperative HRV`s significance in predicting postoperative morbidity and mortality.

## Electronic supplementary material

Below is the link to the electronic supplementary material.

Supplementary Material 1

## Data Availability

No datasets were generated or analysed during the current study.
