# Supplementary material for: Heart rate variability as a predictor of intraoperative autonomic nervous system homeostasis
Source: J Clin Monit Comput. 2024 Jul 13;38(6):1305–13. doi: 10.1007/s10877-024-01190-x (PMC11604806; doi:10.1007/s10877-024-01190-x)
Supplement: Supplementary file 1 — Supplementary Material 1 [file 10877_2024_1190_MOESM1_ESM.docx]

| **Method** | **Parameter** | **Unit** | **Definiton** | **Interpretation** |
| --- | --- | --- | --- | --- |
| Time domain |  |  |  |  |
|  | SDNN | ms | Standard deviation of NN intervals | Reflects the regulatory capacity of the ANS [1] |
|  | RMSSD | ms | Root Mean Square of successive differences | Reflects parasympathetic influence [2] |
| Frequency domain |  |  |  |  |
|  | TP | ms^2^ | Total power | Estimation of overall HRV |
|  | LF | ms^2^ | Low frequency power | Reflects the sympathetic and parasympathetic influence [3] |
|  | HF | ms^2^ | High frequency power | Reflects parasympathetic influence [4] |
|  | LF/HF Ratio | ms^2^ | Ratio of low and high frequency power | Reflects sympathovagal balance [5] |

Supplement Table 1: HRV parameters with units, definitions and interpretation

Abbreviations: NN intervals = normal intervals between adjacent normal R waves in the ECG

[1] Malik M. Heart rate variability: Standards of measurement, physiological interpretation, and clinical use: Task force of the European Society of Cardiology and the North American Society for Pacing and Electrophysiology. Annals of Noninvasive Electrocardiology. 1996;1:151-81.

[2] Kleiger RE, Stein PK, Bigger Jr JT. Heart rate variability: measurement and clinical utility. Annals of Noninvasive Electrocardiology. 2005;10:88-101.

[3] Saul JP. Beat-to-beat variations of heart rate reflect modulation of cardiac autonomic outflow. Physiology. 1990;5:32-7.

[4] Berntson GG, Bigger JT, Jr., Eckberg DL, Grossman P, Kaufmann PG, Malik M, et al. Heart rate variability: origins, methods, and interpretive caveats. Psychophysiology. 1997;34:623-48.

[5] Montano N, Ruscone TG, Porta A, Lombardi F, Pagani M, Malliani A. Power spectrum analysis of heart rate variability to assess the changes in sympathovagal balance during graded orthostatic tilt. Circulation. 1994;90:1826-31.
